# Characterization of oral microbiota in 6–8-month-old small breed dogs

**DOI:** 10.1186/s12917-024-03973-5

**Published:** 2024-04-05

**Authors:** Masahiro Morita, Takayuki Nambu, Ryota Yamasaki, Yoshie Nagai-Yoshioka, Maki Inoue, Tatsuji Nishihara, Toshinori Okinaga, Wataru Ariyoshi

**Affiliations:** 1 Division of Infections and Molecular Biology, Department of Health Promotion, Kyushu Dental University, 2-6-1 Manazuru, Kokurakita-ku, Kitakyushu, Fukuoka 803-8580 Japan; 2 Saki Animal Hospital, 1-19-33, Mukaino, Minami-ku, Fukuoka, 815-0035 Japan; 3 Department of Bacteriology, Osaka Dental University, 8-1, Kuzuha-Hanazono, Hirakata, Osaka 573-1121 Japan; 4 Dental Center for Regional Medical Survey, Kyushu Dental University, 2-6- 1 Manazuru, Kokurakita-ku, Kitakyushu, Fukuoka 803-8580 Japan

**Keywords:** Dog, Puppy, Small breed dog, Plaque, Microbiota, Dental Care, Dentition

## Abstract

**Background:**

Periodontitis is the most common oral disease in dogs, and its progression and severity are influenced by risk factors, such as age and body size. Recent studies have assessed the canine oral microbiota in relation to different stages of periodontitis and niches within the oral cavity. However, knowledge of the bacterial composition at different ages and body sizes, especially in puppies, is limited. This study aimed to characterize the oral microbiota in the healthy gingiva of small breed puppies using next-generation sequencing. Additionally, we assessed the impact of dental care practices and the presence of retained deciduous teeth on the oral microbiota.

**Results:**

In this study, plaque samples were collected from the gingival margin of 20 small breed puppies (age, 6.9 ± 0.6 months). The plaque samples were subjected to next-generation sequencing targeting the V3-V4 region of the 16 S rRNA. The microbiota of the plaque samples was composed mostly of gram-negative bacteria, primarily *Proteobacteria* (54.12%), *Bacteroidetes* (28.79%), and *Fusobacteria* (5.11%). *Moraxella sp.* COT-017, *Capnocytophaga cynodegmi* COT-254, and *Bergeyella zoohelcum* COT-186 were abundant in the oral cavity of the puppies. In contrast, *Neisseria animaloris* were not detected. The high abundance of *Pasteurellaceae* suggests that this genus is characteristic of the oral microbiota in puppies. Dental care practices and the presence of retained deciduous teeth showed no effects on the oral microbiota.

**Conclusions:**

In this study, many bacterial species previously reported to be detected in the normal oral cavity of adult dogs were also detected in 6–8-month-old small breed dogs. On the other hand, some bacterial species were not detected at all, while others were detected in high abundance. These data indicate that the oral microbiota of 6–8-month-old small breed dogs is in the process of maturating in to the adult microbiota and may also have characteristics of the small dog oral microbiota.

**Supplementary Information:**

The online version contains supplementary material available at 10.1186/s12917-024-03973-5.

## Background

Periodontitis, the most common oral disease in dogs, reportedly affects many patients visiting veterinary clinics [1]. Periodontitis is a chronic inflammatory disease induced by dental biofilms, and its progression leads to tooth loss and a significant reduction in the quality of life. *Porphyromonas gingivalis* is considered the major causative bacterium of periodontitis in humans [2]. Periodontitis is induced in specific pathogen-free mice treated with *P. gingivalis* because of changes in the oral microbiota through interactions with commensal bacteria [3]. However, the presence of *P. gingivalis* alone in germ-free mice did not induce periodontitis, leading to the hypothesis that periodontitis is not caused by specific pathogenic bacteria but by dysbiosis, an imbalance of the microbiota [4]. Age, diet, smoking, and systemic disorders, such as diabetes, play roles in the development of periodontitis in humans [5]. Similarly, age, breed, and weight are risk factors for periodontitis in dogs [6]. In addition, small-sized dogs (toy breed dogs), especially Toy Poodles and Miniature Dachshunds, have a higher incidence of periodontitis compared to large-sized dogs [6]. Therefore, evaluation of the various risk factors in addition to microbiota composition is essential to elucidate the etiology of periodontitis.

The human microbiota develops in each part of the body after childbirth, has its own composition and function, and influences host growth and disease development. For example, the gut microbiota during childhood is involved in weight gain [7], development of inflammatory bowel disease [8], and immune function [9].The composition of the oral microbiota during childhood evolves alongside the eruption of deciduous and permanent teeth [10, 11]. In addition, it influences weight gain in early childhood [12] and is associated with diseases, such as dental caries [13] and acute pediatric appendicitis [14]. Therefore, the microbiota during childhood is a notable factor that influences future health.

Various studies have been conducted on microbiota composition and diversity in puppies. Many reports have been published on the gut microbiota, revealing that factors in the mother dog and the environment have a significant effect on the composition of the puppy’s gut microbiota [15]. However, most studies on the canine oral microbiota have been conducted in adult dogs, with reports evaluating changes in microbial composition with the progression of periodontitis [16–19], comparison of different niches such as the supragingival margin, subgingival margin, and buccal mucosa [20, 21], and the influence of diet and dental care [22, 23]. However, there have been no previous studies using next-generation sequencing to assess the oral microbiota of puppies.

Investigation of the maturation of the microbiota from puppies to adult dogs is important to understand the characteristics of the oral microbiota associated with oral and systemic diseases in dogs. Therefore, this study aimed to characterize the oral microbiota of small breed dogs aged 6–8 months and evaluate the effects of dental care practices and the presence of retained deciduous teeth on the oral microbiota.

## Results

### Study cohort

20 dogs (11 female, 9 male) were enrolled in this study. Their average age was 6.9 months (range, 6 to 8 months), and their average weight was 5.0 kg (range, 2.1 to 9.8 kg) (Table 1). The dogs represented 9 different breeds: Miniature Schnauzer (*n* = 4); Toy Poodle, Shiba Inu, and French bulldog (*n* = 3 each); Miniature Dachshund and Chihuahua (*n* = 2 each); and Pekingese, American Cocker Spaniel, and Pomeranian (*n* = 1 each). All dogs had received vaccination and had no history of antibiotic or anti-inflammatory drug administration within 3 months of the examination date. Spaying or castration was performed under general anesthesia after completion of the oral examination and plaque sampling.

### Questionnaire and oral examination

The results of the questionnaire survey are presented in Table 1. Of the 20 dogs included in the present study, 11 received dental care at home. Home dental care methods included dental chews (*n* = 4), dental wipes (*n* = 3), a combination of dental wipes and chews (*n* = 3), and brushing (*n* = 1). Regarding the type of food, 5 dogs were fed dry food only, and 15 dogs were fed a mixture of dry and wet food. Fresh tap water was provided to all the dogs, and coprophagy was not observed.

Intraoral examination under general anesthesia revealed an average of 39 permanent teeth (range, 28–42). 13 dogs had retained deciduous teeth, including maxillary deciduous canines (*n* = 12), mandibular deciduous canines (*n* = 6), maxillary deciduous incisors (*n* = 6), mandibular deciduous premolars (*n* = 3), maxillary deciduous premolars (*n* = 2), mandibular deciduous incisors (*n* = 1), maxillary deciduous molars (*n* = 1), and mandibular deciduous molars (*n* = 1). Skeletal malocclusion was observed in 9 animals according to the malocclusion classification of the World Small Animal Veterinary Association (WASAVA) Global Dental Guidelines [24]: Class I (*n* = 1), Class II (*n* = 1), and Class III (*n* = 7). No swellig, redness of the gingiva, or abnormal alveolar bone findings were observed on the dental radiographs in any of the dogs. Based on these results, all dogs in this study were diagnosed as having periodontal disease stage 0 (clinically normal), according to the WASAVA Global Dental Guidelines [24].

**Table 1:** Questionnaire survey and intraoral examination in 20 dogs

Dog	Bleeds	Sex	Age (M)	Body weight (kg)	Dental Care	Typeof Food	Total number of teeth	Retained deciduous teeth	Malocclusion
Dog1	Shiba Inu	F	8	5.3	Ch	D, W	38	I, C / I, C	-
Dog2	Pekingese	F	6	3.0	Br	D, W	38	I, C / C	Class II
Dog3	Miniture Dachshund	M	7	6.0	Ch	D	42	C / -	Class II
Dog4	French Bulldog	M	7	9.8	-	D, W	38	- / -	Class III
Dog5	Miniture Shunauzer	M	7	7.1	Ch, Wi	D, W	40	C, M / -	-
Dog6	Toy Poodle	M	7	3.0	-	D, W	38	C / C	-
Dog7	French Bulldog	F	6	6.7	-	D, W	40	- / -	Class III
Dog8	Chihuahua	F	6	2.2	-	D, W	38	- / -	Class II
Dog9	Miniture Shunauzer	F	7	3.7	Ch	D, W	42	- / -	-
Dog10	Cocker Spaniel	F	7	8.6	-	D, W	42	- / -	-
Dog11	Pomeranian	F	7	2.7	-	D, W	40	I, C / -	Class III
Dog12	French Bulldog	F	8	5.2	Wi	D, W	40	C / -	Class III
Dog13	Chihuahua	F	7	2.1	-	D, W	28	C, P / C, P	-
Dog14	Miniture Shunauzer	M	6	4.9	-	D	42	C / -	-
Dog15	Toy Poodle	M	7	5.0	-	D, W	40	I, C, P / C, P	-
Dog16	Shiba Inu	F	7	7.4	Ch	D	34	C / P	Class III
Dog17	Toy Poodle	M	8	2.9	Ch, Wi	D	36	I, C / C	Class III
Dog18	Miniture Dachshund	M	7	4.0	Ch, Wi	D, W	42	- / -	-
Dog19	Miniture Shunauzer	M	7	6.1	Wi	D, W	42	I / M	-
Dog20	Shiba Inu	F	6	5.0	Wi	D	42	- / -	-

F, female; M, male

Wi, wiping with dental sheets; Ch, dental chews; Br, brushing

D, dry food; W, wet food

Retained deciduous teeth are indicated as “maxillary/mandible”: I, incisors; C, canines; P, premolars; M, posterior molars

### Microbial composition in dental plaque

**Fig. 1:**
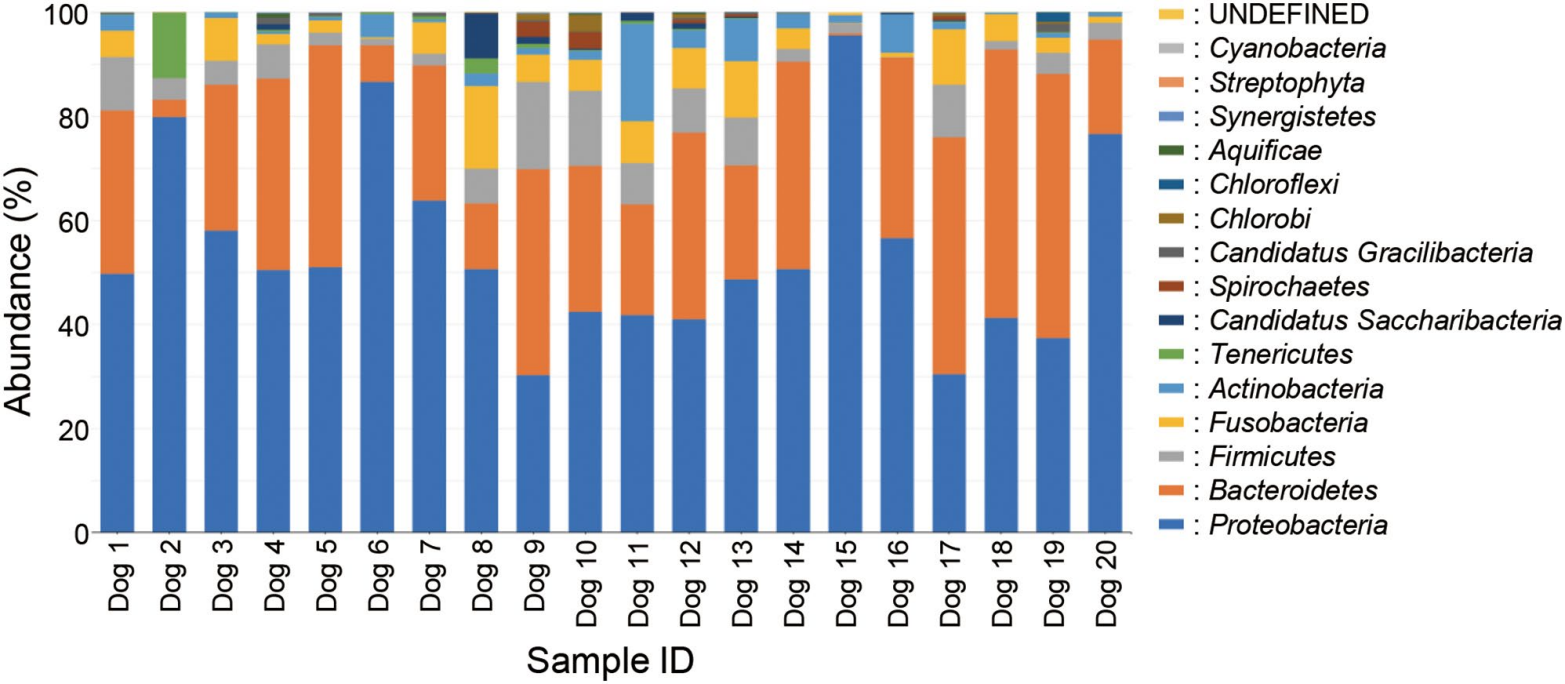
Relative distribution of 16 phyla in the gingival margin plaques of the puppies. The phyla are consistently color-coded and arranged in descending order of abundance, with the most abundant species positioned at the bottom

The total number of reads obtained from the gingival margin plaques collected from the 20 animals in this study was 794,848 for both forward and reverse sequences, with a total of 67,424 reads obtained after denoising using DADA2. The average number of reads per sample was 32,731 (range, 12,080–54,861 reads). A total of 67,424 reads were assigned to 712 amplicon sequence variants (ASVs). The results of the phylum-level database analysis revealed that these 712 ASVs belonged to *Proteobacteria* (54.12%), *Bacteroidetes* (28.79%), *Firmicutes* (5.93%), *Fusobacteri*a (5.11%), *Actinobacteria* (3.06%), *Tenericutes* (0.97%), *Candidatus Saccharibacteria* (0.76%), *Spirochaetes* (0.39%), *Candidatus Gracilibacteria* (0.31%), *Chlorobi* (0.29%), *Chloroflexi* (0.10%), *Aquificae* (0.09%), *Synergistetes* (0.04%), *Streptophyta* (0.003%), *Cyanobacteria* (0.002%), and UNDEFINED (0.03%) (Fig. 1). Of the 20 plaque samples, 16 showed a high abundance of *Proteobacteria*, whereas the remaining 4 samples showed a high abundance of *Bacteroidetes*. 20 species had relative abundances greater than 1% and accounted for 68.5% of the total species (Table 2).*Porphyromonas cangingivalis* COT-109 was the most abundant, representing 10.17% of the total number of sequence reads. *Pasteurellaceae bacterium* COT-271 and *Capnocytophaga cynodegmi* COT-254 were detected in all 20 samples. Other species with high detection rates included *Neisseria canis* COT-137 (19/20), *Moraxella sp.* COT-017 (18/20), *Neisseria dumasiana* (18/20), *Cardiobacterium sp.* COT-238 (18/20), *Pasteurellaceae bacterium* COT-272 (18/20), *Porphyromonas gingivalis* COT-109 (17/20), *Neisseria canis* COT-109 (19/20), *Neisseria sp.* COT-238 (18/20), *Pasteurellaceae bacterium* COT-272 (18/20), *Porphyromonas gingivalis* COT-109 (17/20), *Pasteurellaceae bacterium* COT-080 (17/20), and *Brachymonas sp.* COT-015 (17/20 samples).

**Table 2:** The 20 species present in more than 1% of sequence reads

Species	Percentage identity	Total number of sequence reads	Percentage of total sequence reads (%)	Number of dogs present in 20 dogs
*Porphyromonas cangingivalis* COT-109	100	20,343	10.17	17
*Moraxella* sp. COT-017	100	14,782	7.39	18
*Pasteurellaceae bacterium* COT-271	99.06	11,979	5.99	20
*Pasteurellaceae bacterium* COT-080	100	10,764	5.38	17
*Capnocytophaga cynodegmi* COT-254	100	9268	4.63	20
*Neisseria canis* COT-137	99.77	8155	4.08	19
*Neisseria dumasiana*	100	6996	3.5	18
*Conchiformibius* sp. COT-286	99.77	6806	3.4	16
*Cardiobacterium* sp. COT-238	99.77	5701	2.85	18
*Conchiformibius steedae* COT-280	100	5688	2.84	14
*Pasteurella canis*	100	5602	2.8	16
*Fusobacterium* sp. COT-189	100	5396	2.7	16
*Bergeyella zoohelcum* COT-186	100	4899	2.45	16
*Stenotrophomonas* sp. COT224	100	3824	1.91	16
*Brachymonas* sp. COT-015	99.77	3459	1.73	17
*Moraxella* sp. COT-018	100	3385	1.69	8
*Porphyromonas gingivicanis* COT-022	99.76	2982	1.49	13
*Pasteurellaceae bacterium* COT-272	100	2499	1.25	18
*Porphyromonas gulae* COT-052	100	2435	1.21	10
*Fusobacterium nucleatum*	100	2036	1.02	16

### Gram method stainability and oxygen requirement

A search on PubMed revealed that the ASVs could be classified into 152 genera, including some unclassified genera. A literature search was conducted on the Gram stainability and oxygen requirements of 127 taxable genera. These taxa consisted of 47 Gram-positive and 80 Gram-negative bacteria, with an average of 5.24% Gram-positive and 94.76% Gram-negative bacteria present in each sample (Supplemental Table 1). As for the assessment of oxygen requirement, 49 aerobic, 52 anaerobic, and 26 obligate anaerobic bacteria were identified.The average percentage of aerobic bacteria, anaerobic bacteria, and obligate anaerobic bacteria present in each sample were 55.69%, 22.23%, and 22.08%, respectively (Supplemetal Table 2).

### Statistical analysis

**Fig. 2:**
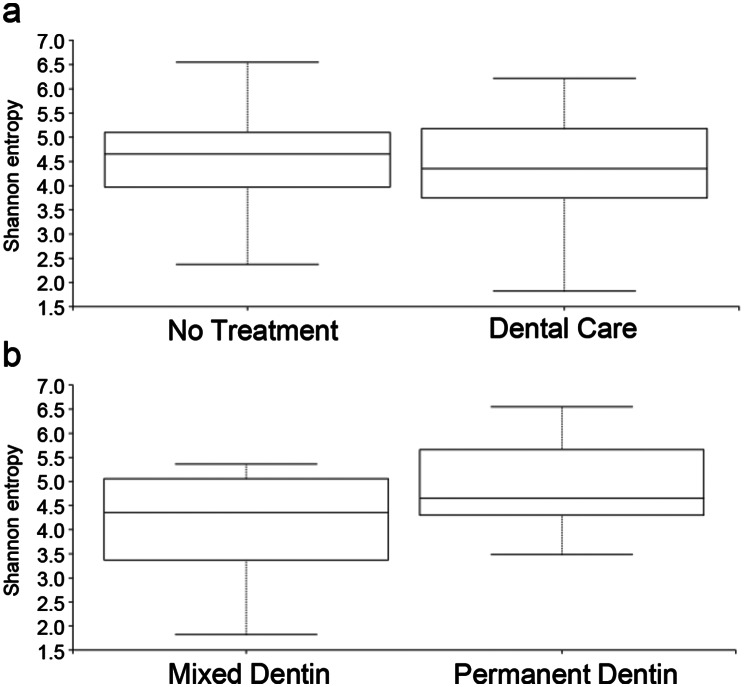
Comparison of the α diversity of the canine plaque samples. Shannon diversity indices with 95% confidence intervals for plaque samples from **(a)** dogs with (Dental care, *n* = 11) or without (No treatment, *n* = 9) dental care (*p* = 0.909) and **(b)** dogs in mixed (Mixed Dentin, *n* = 13) or permanent (Permanent Dentin, *n* = 7) dentition (*p* = 0.285)

Based on the results of the questionnaire survey and oral examination, we statistically analyzed the correlation between home dental care and the presence of retained deciduous teeth with the oral microbiota. 11 dogs received home dental care, whereas 9 dogs did not. 13 dogs had mixed dentition with retained deciduous teeth, 7 dogs had permanent dentition. The results of the α diversity assessment using the Shannon index showed no significant differences in dental care (*p* = 0.909) or dentition (*p* = 0.285) (Fig. 2).

**Fig. 3:**
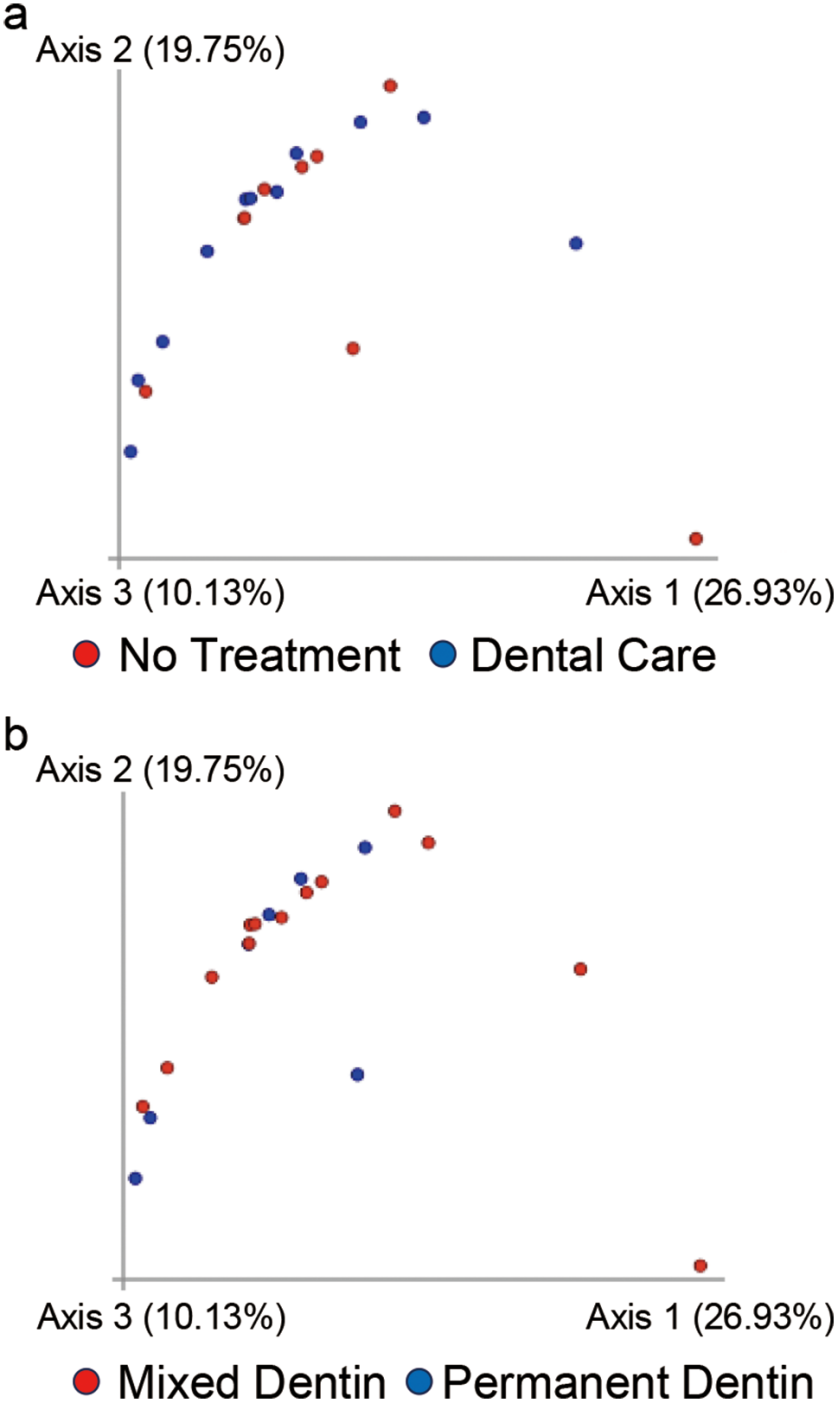
Comparison of the β diversity of the canine plaque samples. Principal coordinate analysis of unweighted UniFrac distances (*p* = 0.001, pairwise permutational analysis of variance) between **(a)** plaque samples from dogs with (Blue plots, *n* = 11) or without (Red plots, *n* = 9) dental care (*p* = 0.858) and **(b)** plaque samples of dogs in mixed (Red plots, *n* = 13) and permanent (Blue plots, *n* = 7) dentition(*p* = 0.834)

**Fig. 4:**
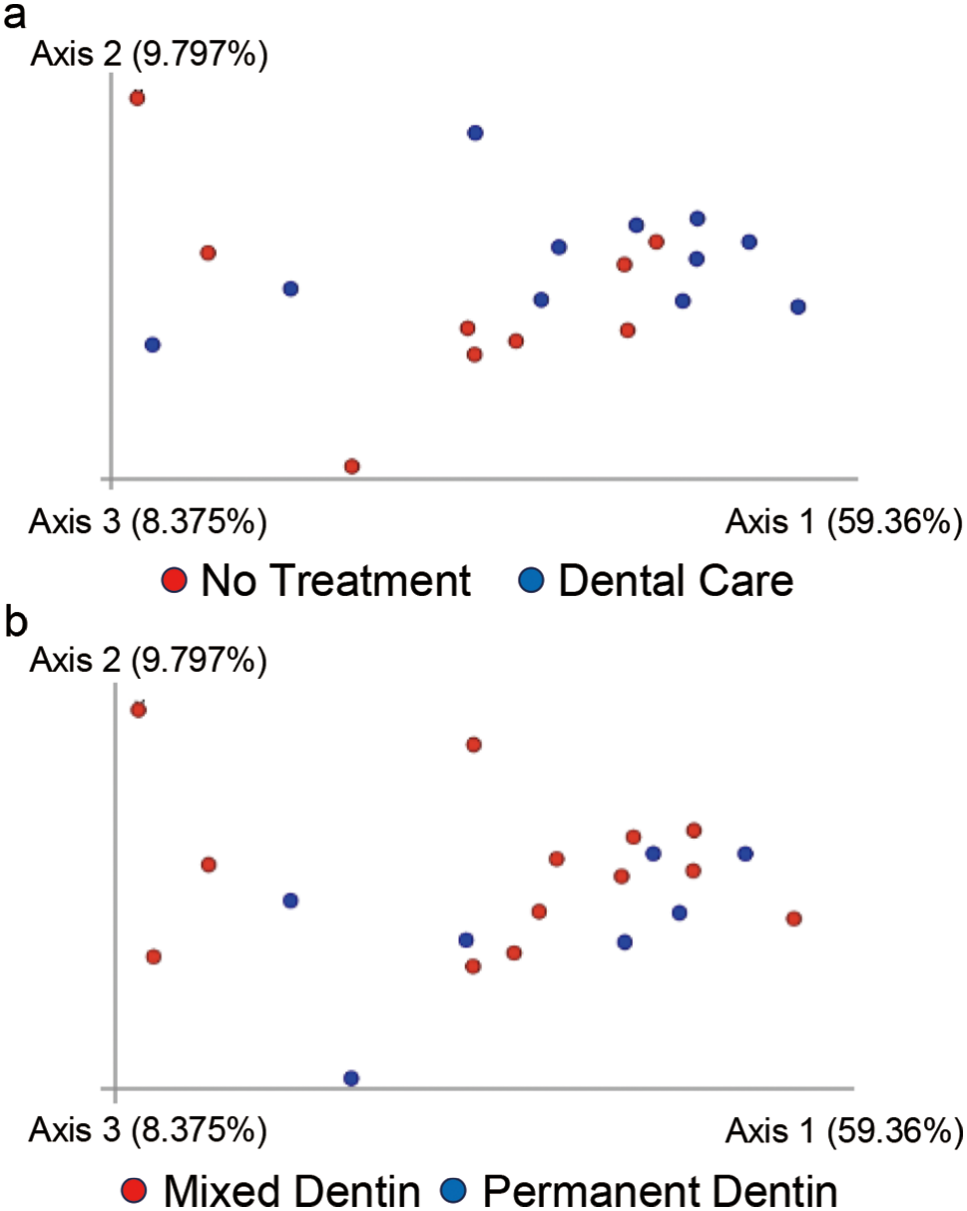
Comparison of the β diversity of canine plaque samples. Principal coordinate analysis of weighted UniFrac distances (*p* = 0.001, pairwise permutational analysis of variance) between **(a)** plaque samples from dogs with (Blue plots, *n* = 11) or without (Red plots, *n* = 9) dental care(*p* = 0.212) and **(b)** plaque samples of dogs in mixed (Red plots, *n* = 13) and permanent (Blue plots, *n* = 7) dentition(*p* = 0.665)

β diversity analysis using both unweighted (Fig. 3) and weighted (Fig. 4) UniFrac distances revealed no significant differences in the microbial community composition for dental care (*p* = 0.858, *p* = 0.212) or dentition (*p* = 0.834, *p* = 0.665).

Analysis of Composition of Microbiomes (ANCOM) was also performed at the order, genus, and species levels. No specific bacteria were significantly altered by home dental care or dentition (data not shown).

## Discussion

Although the uterus was considered sterile in both humans and dogs, recent studies have revealed the presence of microbiota in the placenta and amniotic fluid [25]. Furthermore, microbiota has been identified in the oral cavities of infants [26] and newborn puppies [27]. Differences in the mode of delivery, such as vaginal birth or cesarean section, can affect the oral microbiota in both humans and dogs [28, 29]. Although the birth mode of the puppies in this study was unknown, they showed many similarities to the composition of the oral microbiota in adult dogs. This suggests that the oral microbiota of puppies become similar to that of adult dogs by the age of 6–8 months. Longitudinal plaque samples should be collected from newborn puppies to assess the influence of delivery mode on canine oral microbiota.

Evaluation of the microbiota using next-generation sequencing [16–23] revealed that *Proteobacteria*, *Bacteroidetes*, *Firmicutes*, *Actinobacteria*, and *Fusobacteria* were predominantly present in the oral cavity of dogs, and that their composition changed during oral disease. A previous study in humans comparing subgingival bacteria in healthy individuals and periodontitis patients showed that gram-positive bacteria predominated in the healthy group, whereas gram-negative bacteria were observed predominantly in the periodontitis patients [30]. In contrast, studies on dogs have reported that gram-negative bacteria, mainly *Proteobacteria* and *Bacteroidetes*, predominate in healthy subgingival plaques, whereas gram-positive bacteria, such as *Firmicutes*, increase with the periodontitis progression [16, 18]. In this study, the oral microbiota of the puppies was dominated by *Proteobacteria*, which showed the highest abundance (54.12%). Furthermore, when *Bacteroidetes* (28.25%) and *Fusobacteria* (5.11%) were included, approximately 90% of the microbiota consisted of gram-negative bacteria, which is consistent with the results of a previous study [18].

*P. gingivalis* is a key pathogenic bacterium in human periodontitis [2]. *P. gingivalis* has also been detected in the oral cavity of children [31], and its effect on the oral microbiota during growth has attracted attention. However, the detection rate of *P. gingivalis* in the oral cavity of dogs was low, and its pathogenicity in dogs remains unclear [32, 33]. *Porphyromonas gulae* has been detected in a large number of dogs with periodontitis [34] and exhibits pathogenicity similar to *that of P. gingivalis*, suggesting an important role in the development of periodontitis in dogs [35]. However, *P. gulae* was also present in the healthy oral cavity of dogs [9]. In the present study, *P. gulae* was detected in half of the samples with an abundance of more than 1%. Although no previous studies have evaluated the presence of *P. gulae* in puppies, our study revealed that this bacterium was detected at a high rate in the healthy oral cavity of dogs under 8 months of age. Additional studies are required to assess the presence and quantity of *P. gulae *immediately after birth and evaluateits correlation with the severity of age-related periodontitis. The major *Porphyromonas *present in the oral cavity of dogs was *Porphyromonas cangingivalis* [16, 17]. *P. cangingivalis* possessed heme synthesis and metabolism pathways and was considered to be adaptable to various oral environments [36]. *P. cangingivalis* was frequently detected in both the healthy and periodontitis cases in the oral cavity and was not considered an indicator of oral status [19]. *P. cangingivalis* COT-109 was also the most abundant species in this study (10.17%), indicating that this species adapts to a healthy oral environment under 8 months of age.

In addition to *Porphyromonas*, *Capnocytophaga cynodegmi* COT-254 and *Bergeyella zoohelcum*COT-186 were the other frequently detected Bacteroidetes in this study. These bacteria were detected in healthy gingiva [16], and their abundance decreases with the periodontitis progression [17]. *C. cynodegmi* was present in the oral cavity of 85.8% of adult dogs [37], but it was not found in puppies younger than 6 months of age [38]. In this study, *C. cynodegmi* COT-254 was detected with a high abundance (4.63%) in all 20 dogs aged 6–8 months. This result indicatethat *C. cynodegmi* COT-254 can colonize the oral cavity of dogs by 8 months of age.

*Moraxella* was detected in healthy gingiva [16, 39], especially in supragingival plaques [21]. *Moraxella sp.* COT-017 and COT-018, which were early colonizing species in dental plaques [40], were considered to maintain oral health [17]. In this study, *Moraxella sp.* COT-017 (7.39%) and COT-018 (1.69%) were detected highly abundant in puppies with healthy gingiva, which is consistent with the results of previous studies [17].

*Neisseria* and *Conchiformibius* were detected in high abundance within the Neisseriaceae in this study. *Neisseria*, which was an early plaque-forming bacteria [40], was mainly detected in the healthy oral cavity [16, 17]. In particular, the abundance of *Neisseria shayeganii* COT-090 decreases with periodontitis progression [17]. *Neisseria animaloris* has also been suggested to be a specific marker of a healthy oral cavity [19]. However, in this study, *N. shayeganii* COT-090 was detected at a low abundance of 0.095%, and *N. animaloris* was not identified. In contrast, *Neisseria canis* COT-137 (4.08%) and *Neisseria dumasiana* (3.50%) were highly abundant.Although *N. canis* was often detected in gingivitis [16], and *N. dumasiana* has been isolated from dog-bite wound infection sites [41], there are no reports on the association of these species with canine oral diseases, particularly periodontitis. *Conchifomibius* was detected in high abundance in the healthy oral cavity of dogs 1–7 years of age, and its presence decreased with periodontitis progression [42]. In this study, *Conchifomibius* sp. COT-286 and *Conchifomibius steedae* COT-280 were also detected in high abundance in the oral cavity of 6–8 month of age. The role of *Neisseria* and *Conchiformibius* in a healthy oral cavity needs to be evaluated in the future.

*Fusobacterium nucleatum* co-aggregates with other bacteria and is involved in the plaque-maturation process [43]. In a study of canine early colonizing bacteria of plaque, the abundance of *Fusobacterium sp.* COT-189 was 2.36% and increased 7.63 times from 24 to 48 h after dental cleaning [40]. Therefore, *Fusobacterium sp.* COT-189 was suspected to have functions similar to *F. nucleatum.*In this study, the presence of *Fusobacterium sp.* COT-189 (2.7%) and *F. nucleatum* (1.02%) in the healthy gingival margins of the puppies suggested that these species may play a role in bacterial interactions.

*Pasteurellaceae* COT-080 was detected abundant in the healthy oral cavity [16], and its presence decreases with the periodontitis progression [17]. A study comparing the bacterial composition in different oral niches showed a high abundance of unclassified *Pasteurellaceae* [20]. In addition to the previously unidentified Pasteurellaceae COT-271 and COT-272, various Pasteurellaceae were detected in this study, including Pasteurellaceae COT-080. The abundance of multiple *Pasteurellaceae* may be characteristic of the composition of the healthy microbiota in puppies. *Pasteurella canis* has attracted attention as a pathogen that causes zoonotic diseases in dogs and cats [44]. In this study, *P. canis* was found to be abundant in the healthy oral cavity of the puppies. Notably, zoonotic pathogens are present in the canine oral cavity regardless of age.

Although differences in the microbiota of deciduous [45] and mixed [10] dentition have been reported in humans, the effects of dentition on microbial composition in dogs have not been evaluated.In this study, of the 13 dogs in the group with retained deciduous teeth, 12 dogs had retained maxillary deciduous canines, and these plaque samples were collected from both the maxillary deciduous canines and the permanent canines. Therefore, this sampling method may have contributed to the lack of significant differences in the effects of the retained deciduous teeth on the microbiota.

Daily use of dental chews or brushing has been reported to significantly inhibit plaque and calculus formation, whereas brushing once per week was found to be insufficient [46, 47]. In addition, the oral microbiota of dogs managed using dental chews within the facility showed an increased abundance of bacteria associated with healthy gingiva [22, 47]. Therefore, dental care at the appropriate frequency can be considered to help prevent periodontitis in dogs. In this study, dental care was provided to 11 dogs, of which 7 dogs used dental chews. However, the type of dental chew was not standardized, and the frequency of use varied from daily to weekly. Gauze wiping of the tooth surfaces was performed in 6 dogs, but as with dental chews, the frequency and proficiency differed among dogs.Gauze wiping of the anterior teeth of young children was effective in removing dental plaque [48]. In contrast, in dogs, cleaning teeth with gauze was not effective for removing subgingival plaque, and brushing was recommended for the long-term maintenance of healthy periodontal tissue [24]. Therefore, plaque removal may not have been sufficient in some dogs in the dental care group, which may have contributed to the lack of a difference in oral microbiota composition between the non-dental and dental care groups. Thus, controlling for dental care methods, frequency, and proficiency was necessary before conducting the survey.

The limitations of this study include the small sample size and the bias caused by daily dietary habits and lifestyle. In addition, plaque samples have been collected from small dogs of various breeds, but they are insufficient for the characteristics of the oral microbiota of specific breed. Furthermore, comparative validation of brachycephalic breed dogs with a genetically skeletal malocclusion and dogs with a normal occlusion may contribute to the understanding of periodontal disease risk in small breed dogs. Future studies should aim to collect a larger number of plaque samples and conduct a longitudinal search starting from birth to verify detailed changes in the oral microbiota of each breed dogs.

## Conclusion

This novel study characterized the oral microbiota of small breed dogs aged 6–8 months. Assessment of the oral microbiota in puppies is important for understanding the changes in the microbiota with aging.In addition, small breed dogs show a high incidence of periodontitis and require early diagnosis and therapeutic intervention. In this study, *Moraxella* sp. COT-017, *C. cynodegmi* COT-254, and *B. zoohelcum* COT-186, which were in healthy oral cavity of adult dogs, were present in the gingiva of the puppies. Furthermore, the high abundance of *Pasteurellaceae* in the oral cavity of the puppies was a novel and interesting finding. In contrast, *Neisseria animaloris* which were in the healthy gingiva of adult dogs, were not detected in the puppies in the present study. These data suggest that the microbiota present in the healthy oral cavity at 6–8 months of age reflects the process of maturation.

## Methods

### Study design

20 healthy dogs with a body weight of ≤ 10 kg who visited Saki Veterinary Hospital (Fukuoka, Japan) between June 2021 and October 2021 to undergo spaying or castration surgery were included in this study. Informed consent for the questionnaire survey, oral examination, and plaque collection was obtained from all owners. All procedures were approved by the Animal Care and Use Committee of Kyushu Dental University (#21 − 06).

### Survey of owners intraoral examination of dogs.

Dog owners who participated in this study were interviewed regarding home dental care (dental sheet, dental chew, or brushing), type of food (dry or wet), coprophagia, and medication history (Supplemental Table 3). Intraoral examination and plaque sampling were performed under general anesthesia at the time of spaying or castration surgery. The owners were instructed to start fasting the dogs 12 h prior to plaque collection. During the oral examination, the total number of teeth, total number of retained deciduous teeth, presence of malocclusion, and gingival swelling or redness were evaluated, and dental radiographs were obtained. The results of oral examination were recorded in the sample dental chart of the WASAVA Global Dental Guidelines [24]. Periodontitis was classified according to the WASAVA Global Dental Guidelines [24]. Before the oral examination, dental plaque samples were collected from the gingival margins of the bilateral maxillary canine and fourth premolar teeth using a sterile swab (OMINIGEN-ORAL; DNA Genotek Inc., Ottawa, Canada). In cases with retained deciduous teeth at the sampling site, plaque samples were collected from the gingival margins of both deciduous and permanent teeth. Plaque samples were collected with a sterile swab, placed in storage tubes, shaken 10 times, and stored at 25–28°Cof room temperature.

### DNA extraction

Collected dog plaque samples were mixed with Ready-Lyse Lysozyme Solution (1250 U/sample) (Epicentre, Madison, WI) and incubated at 37 °C for 18 h. Genomic DNA was extracted from the samples using the MasterPure™ Complete DNA and RNA Purification Kit (Epicentre, Madison, WI). The extracted DNA pellets were dissolved in 50 µL of TE buffer. DNA concentration was measured using a NanoDrop 2000 spectrophotometer (Thermo Fisher Scientific, Waltham, MA, USAs). All samples were stored at -40°Cuntil further analysis.

### Amplification of the 16 S rRNA gene

DNA extracted from the plaque was amplified from the V3-V4 region of the bacterial 16 S rRNA using polymerase chain reaction (PCR). The universal primers for 16 S rRNA were 341 F, 5′-CCT ACG GNG GCW GCA G-3′, and 805R, 5′-GAC TAC HVG GTA TCT AAT CC-3′. PCR was performed under the following conditions: initial denaturation at 96 °C for 2 min, followed by 25 cycles of denaturation at 96 °C for 30 s, annealing at 55 °C for 45 s, and elongation at 72 °C for 1 min, with a final extension at 72 °C for 10 min. Sequencing was performed using a MiSeq platform (Illumina, San Diego, CA, USA).

### Sequence data processing

The processed sequences of each sample were analyzed using QIIME2 [49] in a Docker environment. The forward and reverse reads of each sample were processed using DADA2 [50] and included filtering, merging, and chimera removal steps. ASVs were taxonomically assigned to the Silva SSU database release 138 [51] and the Canine Oral Microbiome Database [52] using BLASTN for phylogenetic analysis.

### Detection of gram method stainability and oxygen requirement

The genus names, excluding rare ASVs obtained by searching PubMed (https://pubmed.ncbi.nlm.nih.gov), were classified as “gram-positive” or “gram-negative” based on literature searches. A similar approach was used to categorize the bacteria as “aerobic,” “anaerobic,” and “facultative anaerobic” based on their oxygen demand. The number of genera and the percentage of each genus present in each sample were calculated.

### Statistical analysis

Statistical analyses were performed using QIIME2 (Ver2022.2.0). α diversity analysis, which indicates the diversity within a sample, was performed using the Shannon index. β diversity analysis, which indicates differences between samples, was performed using principal coordinate analysis of unweighted and weighted UniFrac distances. Additionally, significant differences in the prevalence of individual bacterial species among the sample groups were determined by ANCOM [53].

### Electronic supplementary material

Below is the link to the electronic supplementary material.

Supplementary Material 1

Supplementary Material 2

Supplementary Material 3

## Data Availability

All raw sequence data generated in this study were obtained from the DNA Data Bank of Japan under the accession number DRA017124// (http://www.ddbj.nig.ac.jp/, https://ddbj.nig.ac.jp/search, accessed on 26 September 2023).

## Abbreviations

**ANCOM**: Analysis of microbiome composition
**ASV**: Amplicon sequence variants
**COT**: Canine Oral Taxon
**DNA**: Deoxyribonucleic acid
**PCR**: Polymerase chain reaction
**PD0**: Periodontal disease stage 0
**WASAVA**: World Small Animal Veterinary Association
